# Bromide of Potassium in Its Effect on Children

**Published:** 1870-10

**Authors:** 


Bromide of Potassium in Its Effect on Children.—E. Montard-Martin, of the Beaujou Hospital, France, reports a number of cases to show the effects of this drug on infants and small children. It is invaluable in all cases of dentition, where there are aggravating nervous symptoms, and it is improper to give opium. In sleeplessness of infants, in cough purely spasmodic, he gives it in doses of $\frac{3}{4}$ gr. once or twice a day to children from three months to a year old. In purely nervous cough it is equally indicated; but it should not be given in diarrhœa, as it increases this difficulty. In excessive erethism of any sort, and especially in those cases of boys where there is almost constant erection of the penis, it works most happily.

When administered to infants, the breast should be given at once afterwards.—*New York Medical Journal.*

